# Endobronchial metastasis of hepatocellular carcinoma treated with Lenvatinib: A case report

**DOI:** 10.1002/rcr2.1208

**Published:** 2023-08-22

**Authors:** Shota Takei, Akihiro Yoshimura, Chie Yamamoto, Seita Kataoka, Nobutaka Kataoka, Kenji Morimoto, Masahiro Iwasaku, Shinsaku Tokuda, Tadaaki Yamada, Takayuki Takeda, Koichi Takayama

**Affiliations:** ^1^ Department of Pulmonary Medicine Kyoto Prefectural University of Medicine Kyoto Japan; ^2^ Department of Respiratory Medicine Japanese Red Cross Kyoto Daini Hospital Kyoto Japan; ^3^ Department of Infectious Disease Kyoto Prefectural University of Medicine Kyoto Japan; ^4^ Department of Molecular Gastroenterology and Hepatology Kyoto Prefectural University of Medicine Kyoto Japan

**Keywords:** bronchoscopy, endobronchial metastasis, hepatocellular carcinoma, lenvatinib

## Abstract

Endobronchial metastasis (EBM) of hepatocellular carcinoma (HCC) is rare though HCC often metastasizes to the lungs. In this case report, a 74‐year‐old man with a history of HCC with chronic hepatitis C experienced hemoptysis and a dry cough. During immunotherapy for postoperative recurrent HCC, chest computed tomography (CT) revealed soft tissue shadows in the right upper and lower lobe bronchi, and we pathologically diagnosed as EBM of HCC using bronchoscopy. Although the prognosis of HCC with EBM is poor, lenvatinib, a tyrosine kinase inhibitor, was administered and resulted in improved symptoms, decreased tumour markers, and reduced EBM shadows on chest CT scans. To the best of our knowledge, this is the first case of lenvatinib monotherapy for EBM of HCC. It is important to perform a bronchoscopy for early diagnosis of EBM, followed by lenvatinib treatment.

## Graphical abstract

Chest CT showed soft tissue shadows in the right B3b. Fiberoptic bronchoscopy revealed endobronchial tumours in the right B3b and histopathological examination of a biopsy specimen confirmed HCC metastasis.

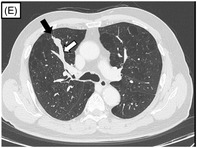

## INTRODUCTION

Lungs are the most frequent metastatic sites for hepatocellular carcinoma (HCC).^1^ In contrast, endobronchial metastasis (EBM) of HCC is rare compared to other malignancies.^2^ In this case report, we present a patient diagnosed with EBM of HCC using bronchoscopy. Although the prognosis of EBM of HCC has been reported to be worse,^2^ the patient's EBM shadow was reduced on chest computed tomography (CT) following lenvatinib administration.

## CASE REPORT

**FIGURE 1:**
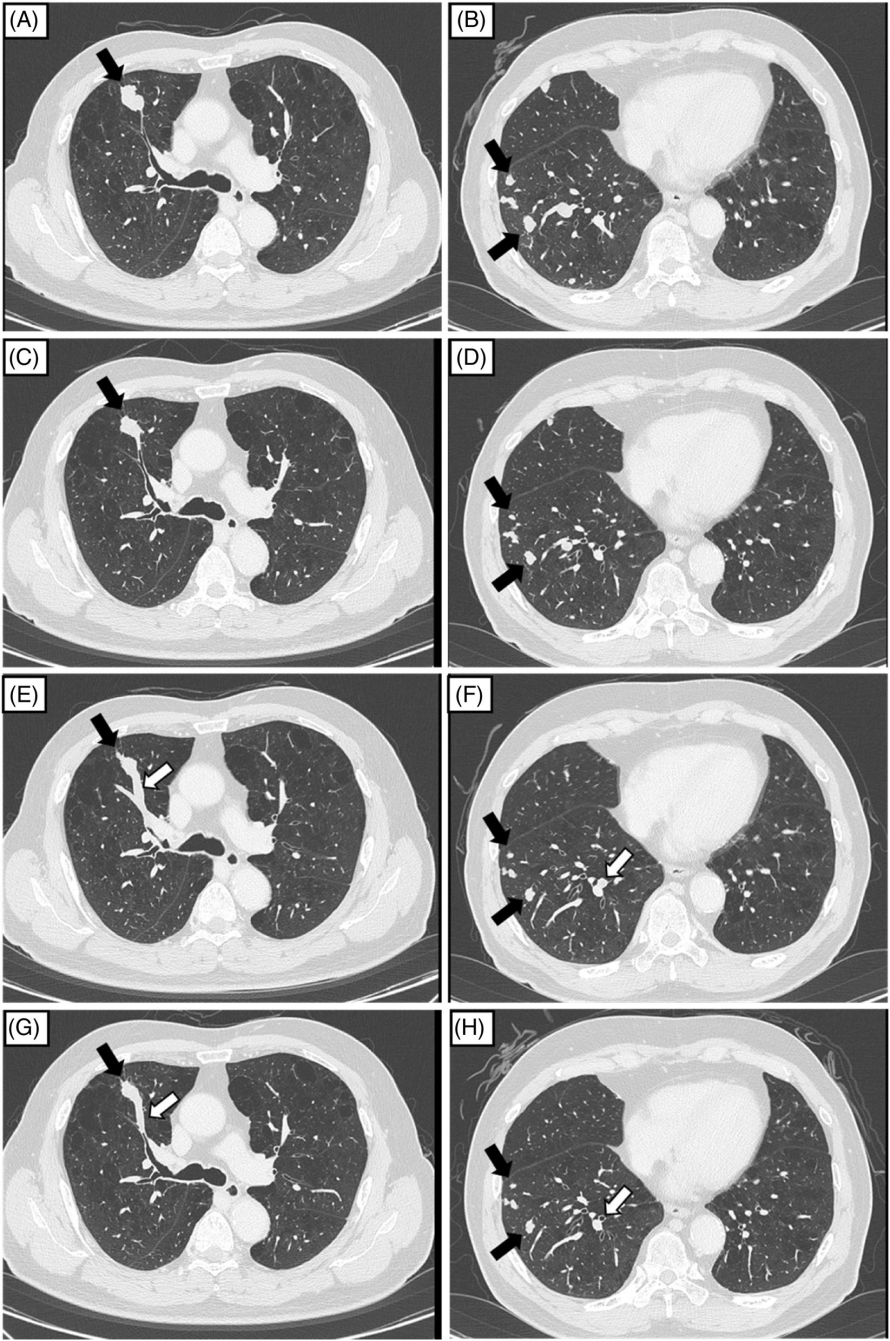
Chest computed tomography (CT) revealed that bilateral multiple lung metastases (black arrows) were found in all lung fields, 2 years after liver lobectomy (A, B). After four cycles of combination therapy with atezolizumab and bevacizumab, multiple lung metastases were reduced (C, D). After 23 cycles of the combination therapy, chest CT showed that soft tissue shadows (white arrow) appeared within the right upper and lower bronchi. However, lung metastases were maintained without an increase in the size of the existing lesions or the appearance of new lesions (E, F). After lenvatinib induction, endobronchial metastases were reduced (G, H).

A 74‐year‐old man presented with hemoptysis and a dry cough. He had a history of HCC with chronic hepatitis C, which was treated with liver lobectomy 2 years before presentation. Histological examination of the resected liver specimen showed a tumour, 90 mm in diameter, located in the posterior segment, and a poorly differentiated HCC with portal vein invasion. Five months postoperatively, serum protein induced by vitamin K absence or antagonist‐II (PIVKA‐II) levels increased, and chest CT scans revealed multiple nodules bilaterally in the lungs (Figure 1A, B).

**FIGURE 2:**
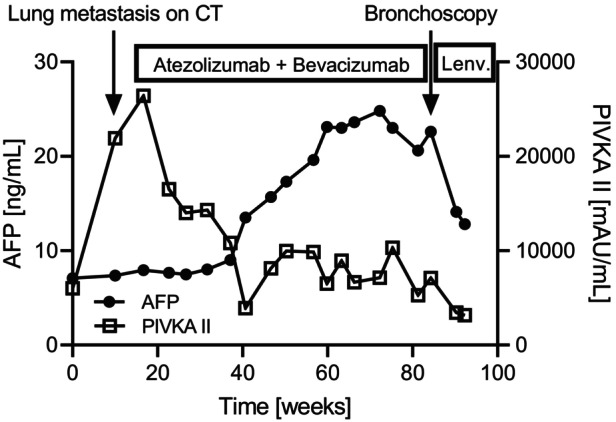
Clinical course. Five months after hepatic lobectomy, serum PIVKA‐II levels increased, and a chest CT scan showed multiple nodules bilaterally in the lungs. The patient was diagnosed with lung metastasis of hepatocellular carcinoma (HCC), and underwent combination therapy with atezolizumab and bevacizumab. After introducing the combination therapy, serum PIVKA‐II levels decreased long‐term. However, serum AFP levels gradually increased, and bronchoscopy examination revealed endobronchial metastasis of HCC. After the induction of lenvatinib, serum AFP levels decreased. AFP, alpha‐fetoprotein; PIVKA‐II, protein induced by vitamin K absence or antagonist‐II; CT, computed tomography; Lenv, lenvatinib.

The patient was clinically diagnosed with an early recurrence of HCC, and underwent salvage chemotherapy with atezolizumab (1200 mg) and bevacizumab (15 mg/kg). After four cycles of combination therapy with atezolizumab and bevacizumab, the serum PIVKA‐II levels decreased, lung metastases were reduced on chest CT, and the patient achieved a partial response (Figure 1C, D). However, after 23 cycles of combination therapy, the patient reported hemoptysis and dry cough. The chest CT revealed soft tissue shadows in the right upper and lower lobe bronchi, although the lung metastases did not increase (Figure 1E, F). On admission, coarse crackles were audible in the right lower lobe on physical examination. Although the serum PIVKA‐II levels did not increase, serum alpha‐fetoprotein (AFP) levels gradually increased (Figure 2).

**FIGURE 3:**
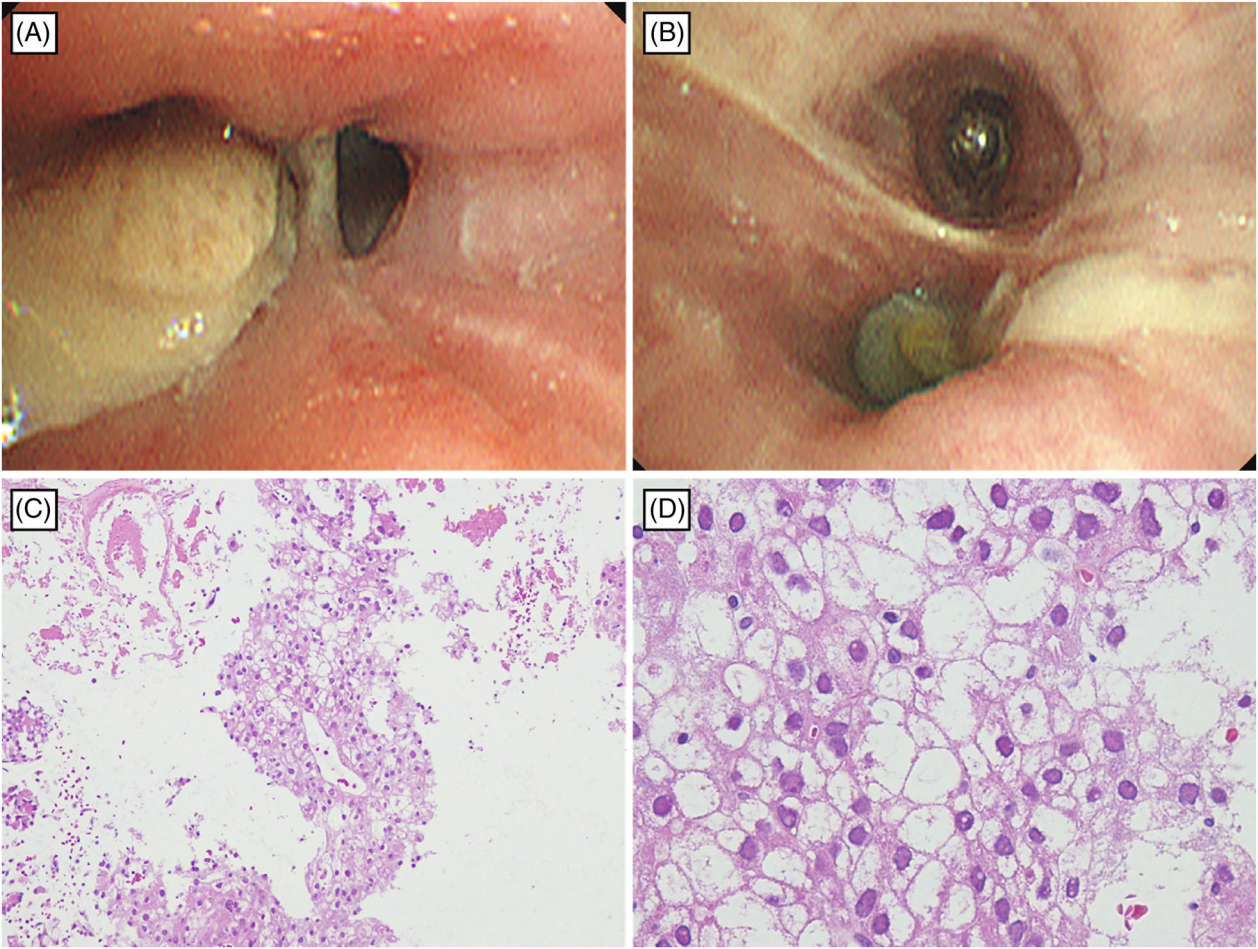
Bronchoscopy examination showed peripheral airway obstruction due to tumours observed in the right B^3^b (A) and B^10^ (B). Histological examination of a transbronchial biopsy specimen revealed atypical cells with eosinophilic cytoplasm, forming a cobblestone‐like cluster. It presented findings similar to those of previous studies on hepatocellular carcinoma (Haematoxylin and Eosin staining, C: ×100, D: ×400).

For careful examination of hemoptysis, fiberoptic bronchoscopy was performed, which revealed endobronchial tumours in the right B^3b^ and B^10^. Histopathological examination of a biopsy specimen in the right B^3b^ confirmed HCC metastasis (Figure 3). Bronchoalveolar lavage fluid cultures were negative for bacteria, mycobacteria and fungi. Therefore, we pathologically diagnosed the patient with EBM of HCC. After thoroughly explaining the potential bleeding risks associated with lenvatinib, we obtained the patient's informed consent, and then administered lenvatinib (12 mg). Lenvatinib administration quickly improved hemoptysis, decreased tumour marker levels, and reduced EBM shadows as shown by chest CT (Figure 1G, H).

## DISCUSSION

Hepatocellular carcinoma is a malignant tumour that usually develops during the course of chronic liver disease, especially related to the hepatitis B virus or hepatitis C virus. Among the several recurrence patterns after radical resection, HCC often metastasizes to the lungs^1^; however, only approximately 10 cases of EBM of HCC have been reported.^2^

The concept of EBM was categorized into the following four developmental modes by Kiryu^3^: type I, direct metastasis to the bronchus; type II, bronchial invasion by a parenchymal lesion; type III, bronchial invasion by mediastinal or hilar lymph node metastasis; and type IV, peripheral lesions that extend along the proximal bronchus. The authors mentioned that the incidence and prognosis of the EBM varied by type; type IV was the most frequent (56.3%), and the prognosis of type III was significantly worse.^3^ The EBM in our patient was classified as type IV according to the EBM criteria. Moreover, as previously reported, EBM was often observed in the right lung, although the reason for this is unclear. The patient also had EBM of HCC in the right lung. The EBM of HCC has a poor prognosis, and the efficacy of some therapies, such as chemotherapy and radiotherapy, is insufficient for HCC patients with EBM.^2^

To the best of our knowledge, this is the first case of lenvatinib monotherapy for EBM of HCC, in which lenvatinib worked quickly and for more than 6 months. Lenvatinib administration not only improved hemoptysis but also decreased serum tumour marker levels in our patient. The most commonly used tumour marker for HCC in clinical setting is serum AFP, a fetal form of albumin, and serum PIVKA‐II, an abnormal prothrombin precursor without coagulation activity. Since serum PIVKA‐II levels do not correlate with serum AFP levels, simultaneous measurement of these tumour makers has been recommended for follow‐up of HCC and evaluation of therapeutic efficacy.^4^ Consistent with the previous report, in this patient, PIVKA‐II was useful at diagnosis and AFP was useful at relapse and for the therapeutic effect of lenvatinib.

Lenvatinib is a multi‐targeted tyrosine kinase inhibitor (TKI) that targets vascular endothelial growth factor (VEGF) receptors 1–3 and fibroblast growth factor (FGF) receptors 1–4, and has been reported to be effective for unresectable HCC patients after resistance to combination therapy with atezolizumab and bevacizumab.^4^ Therefore, as in our patient, lenvatinib is the second‐line treatment of choice for its efficacy, after resistance to the combination therapy with atezolizumab and bevacizumab. While the anti‐tumour effect of lenvatinib has been reported to be immediate,^5^ the Norton–Simon hypothesis states that as a tumour grows larger, its growth rate increases; however, when it is smaller, its growth rate slows down and becomes more sensitive to chemotherapy.^6^ Therefore, it is important to perform bronchoscopic examinations early, even if the shadow resembles a mucous plug at first glance, as in the present case, and to treat the patient with lenvatinib without missing the therapeutic opportunity.

In conclusion, we encountered a case of EBM, diagnosed via bronchoscopic examination in a patient with HCC. The patient's EBM shadow was reduced following lenvatinib treatment. Therefore, for early diagnosis of EBM, it is important to perform a bronchoscopy followed by lenvatinib treatment.

## AUTHOR CONTRIBUTIONS

All authors contributed to the conception of the case report. Shota Takei, Chie Yamamoto, and Nobutaka Kataoka performed bronchoscopy. Shota Takei, Akihiro Yoshimura, and Seita Kataoka participated in patient care. Shota Takei and Akihiro Yoshimura drafted the manuscript, and all other authors contributed to the critical review of the article for important intellectual content. All authors read and approved the final manuscript.

## CONFLICT OF INTEREST STATEMENT

None declared.

## ETHICS STATEMENT

The authors declare that appropriate written informed consent was obtained for the publication of this manuscript and accompanying images.

## Data Availability

The data that support the findings of this study are available from the corresponding author upon reasonable request.

## References

[1] Uchino K , Tateishi R , Shiina S , Kanda M , Masuzaki R , Kondo Y , et al. Hepatocellular carcinoma with extrahepatic metastasis: clinical features and prognostic factors. Cancer. 2011;117:4475–4483. 21437884 10.1002/cncr.25960

[2] Cheung FP , Russell PA , Alam NZ , Wright GM . Endobronchial metastases from hepatocellular carcinoma: a case report. Asian Cardiovasc Thorac Ann. 2019;27:703–706. 31522515 10.1177/0218492319876493

[3] Kiryu T , Hoshi H , Matsui E , Iwata H , Kokubo M , Shimokawa K , et al. Endotracheal/endobronchial metastases: clinicopathologic study with special reference to developmental modes. Chest. 2001;119:768–775. 11243955 10.1378/chest.119.3.768

[4] Ishii M , Gama H , Chida N , Ueno Y , Shinzawa H , Takagi T , et al. Simultaneous measurements of serum alpha‐fetoprotein and protein induced by vitamin K absence for detecting hepatocellular carcinoma. South Tohoku District Study Group. Am J Gastroenterol. 2000;95:1036–1040. 10.1111/j.1572-0241.2000.01978.x 10763956

[5] Aoki T , Kudo M , Ueshima K , Morita M , Chishina H , Takita M , et al. Exploratory analysis of Lenvatinib therapy in patients with unresectable hepatocellular carcinoma who have failed prior PD‐1/PD‐L1 checkpoint blockade. Cancers (Basel). 2020;12:3048. 33092011 10.3390/cancers12103048 PMC7590172

[6] Norton L , Simon R . The Norton‐Simon hypothesis revisited. Cancer Treat Rep. 1986;70:163–169. 3510732

